# Resurgence of Respiratory Syncytial Virus in the Summer of 2021 in Denmark—a Large out-of-season Epidemic Affecting Older Children

**DOI:** 10.1093/ofid/ofae069

**Published:** 2024-02-05

**Authors:** Frederikke Kristensen Lomholt, Hanne-Dorthe Emborg, Sarah Kristine Nørgaard, Jens Nielsen, Charlotte Munkstrup, Karina Lauenborg Møller, Jesper Schak Krog, Ramona Trebbien, Lasse Skafte Vestergaard

**Affiliations:** Department of Infections Disease Epidemiology and Prevention, Statens Serum Institut, Copenhagen, Denmark; Department of Infections Disease Epidemiology and Prevention, Statens Serum Institut, Copenhagen, Denmark; Department of Infections Disease Epidemiology and Prevention, Statens Serum Institut, Copenhagen, Denmark; Department of Infections Disease Epidemiology and Prevention, Statens Serum Institut, Copenhagen, Denmark; Department of Infections Disease Epidemiology and Prevention, Statens Serum Institut, Copenhagen, Denmark; Division of Infectious Disease Preparedness, Statens Serum Institut, Copenhagen, Denmark; Department of Virus and Microbiological Special Diagnostics, Statens Serum Institut, Copenhagen, Denmark; Department of Virus and Microbiological Special Diagnostics, Statens Serum Institut, Copenhagen, Denmark; Department of Infections Disease Epidemiology and Prevention, Statens Serum Institut, Copenhagen, Denmark

**Keywords:** epidemiology, out-of-season epidemic, register-based study, respiratory syncytial virus (RSV), surveillance

## Abstract

**Background:**

When coronavirus disease 2019 (COVID-19) restrictions were lifted in Denmark in the spring of 2021, a surge in respiratory syncytial virus (RSV) cases followed, causing a large out-of-season epidemic. This study aims to investigate the summer epidemic compared with 3 typical pre-COVID-19 RSV winter seasons using Danish registers to identify RSV cases, RSV-related admissions, and use of intensive care treatment.

**Methods:**

Incidence rates (IR) per 1000 person-years for RSV cases, RSV-related admissions, and intensive care treatment were calculated with 95% confidence interval (CI) for each season, stratified by age groups and incidence rate ratios (IRR) with 95% CI were calculated to compare the summer epidemic with the winter season for 2019-2020.

**Results:**

In the summer epidemic, the IR of RSV cases and admissions exceeded previous winter seasons for all age groups. The highest increases in IRs were seen among children aged 2 to 3 years and 4 to 5 years. The IRR of cases were 4.6 (95% CI, 4.1-5.2) and 3.3 (2.6-4.2) and the IRR of admissions were 3.3 (2.7-4.2) and 3.8 (2.3-6.5) in the 2 age groups, respectively, when compared with the winter season 2019-2020.

**Conclusions:**

Likely because of immunity debt following COVID-19 restrictions, the summer epidemic was significantly larger than previous winter seasons, most markedly among children aged 2 to 3 and 4 to 5 years but had a similar disease severity spectrum.

Respiratory syncytial virus (RSV) is a virus that can cause upper and lower respiratory tract infections in both children and adults. In most cases, the virus causes mild infection but infants are especially at increased risk of developing severe disease [1]. RSV is the most common cause of severe respiratory infection in infants. By the age of 12 months, approximately two thirds of all children have been infected with RSV and by the age of 2 years, almost all children have been infected with RSV [2, 3]. In Denmark, the number of RSV cases usually follows a distinct annual pattern in which the number of cases increases in November and December and peaks in January or February [4]. The seasonality of RSV before the coronavirus disease 2019 (COVID-19) pandemic showed consistency between countries with start, end, and peak in activity only differing by 1 to 3 weeks, and surveillance data from Denmark show similar results [4, 5].

During the COVID-19 pandemic, Denmark implemented social restrictions and sanitary measures to reduce transmission of severe acute respiratory syndrome coronavirus 2 (SARS-CoV-2) [6]. Consequently, a drop in other respiratory infections followed [7, 8], and the expected winter season of RSV in 2020-2021 did not appear. However, as the social and sanitary measures were lifted during the spring and summer of 2021, a surge in RSV cases was detected in Denmark, leading to a dramatic summer epidemic in which the number of cases exceeded the number of cases in any previous season. Furthermore, Danish pediatric hospital departments reported an unusual high number of RSV-related admissions among children. Similar unusual out-of-season RSV outbreaks were reported in several other countries [9–13].

It has been hypothesized that the decrease in circulating respiratory pathogens because of restrictions have reduced the overall immunity in society and therefore leaving a higher proportion of people susceptible to infections, and thus, possibly leading to higher peaks in following seasons for respiratory infections [14, 15]. Especially for RSV, in which early childhood infections are so common, the absence of the expected winter season might have left a high number of children immunologically naïve in the spring of 2021. The aim of this study is to describe the epidemiology of RSV in Denmark from 2017 to 2022 using national registers and to compare the summer epidemic with previous seasons.

## METHODS

### Study Period

A register-based study was conducted covering the period from week 21, 2017, to week 20, 2022. A surveillance season was defined as starting in week 21 spanning to week 20 in the following year.

### Study Population

The entire Danish population of approximately 6 million people was included in the study by using the Danish Civil Registration System (DCRS) that holds information on all Danish residents [16]. The population was defined for each season as residents living in Denmark during the particular season. RSV test results were extracted from the Danish Microbiology Database (MiBa) [17]. MiBa is a national database that collects information on microbiological diagnostic tests and test results, both positive and negative, from all departments of clinical microbiology in Denmark, which include tests performed during inpatient and outpatient hospital contacts as well as tests performed at general practitioner level. MiBa does not include information on indication for testing. A case of RSV was defined as any individual who tested positive for RSV at least once by polymerase chain reaction test. Only the first positive test in each surveillance season was included in the analyses, but an individual could be identified as an RSV case in more than 1 season.

### Covariates

The DCRS holds information on all Danish residents using a 10-digit unique personal identifier. From the DCRS, information on date of birth, vital status, sex, date of death, date of immigration and emigration, and place of residence were extracted for the entire study population.

Age was defined as age at sample date and age groups were defined based on the risk of severe disease from an RSV infection [4]: 0 to 2 months; 3 to 5 months; 6 to 23 months; 2 to 3 years; 4 to 5 years; 6 to 14 years; 15 to 44 years; 45 to 64 years; and ≥65 years of age.

Data on RSV-related hospital contacts and possible need for intensive care treatment (ICT) were extracted from the Danish National Patient Registry, which contains information on all contacts to a Danish hospital including contacts to emergency departments, psychiatric hospitals, and outpatient clinics [18]. For each contact, 1 primary and 1 or more optional secondary diagnosis are provided. In addition, procedure codes (eg, mechanical ventilation) are registered in the Danish National Patient Registry. An RSV-related hospital admission was defined as a hospital contact lasting at least 12 hours, where the RSV-positive test was sampled either during the admission or up to 4 days before the admission. An ICT admission was defined as an RSV admission in which at least 1 ICT procedure was performed during the admission (see Supplementary Table 1 for the list of ICT procedure codes).

The 10-digit unique personal identifier was used to link data from the different registers.

### Statistical Analyses

Only 1 sample per person per week was included and the positive test was chosen if both positive and negative tests were available. For persons with a positive test (cases), only negative tests up until the first positive test in that surveillance season were included. The seasonal number of RSV cases, RSV tests, RSV-related admissions, and RSV-related ICT were calculated in total and by age group. The weekly number of cases and RSV tests was calculated for each season, and the weekly percent positive was calculated as the weekly number of RSV-positive tests divided by the weekly number of RSV tests.

For each age group, seasonal case incidence, incidence rate of RSV-related admissions, and incidence rate of RSV-related ICT admissions were calculated per 1000 person-years with 95% confidence interval (CI). Person-time was calculated for each individual in each season as described in Supplementary Figure 1. In addition, incidence rates of cases, admissions, and ICT by age group in the season of the summer epidemic, 2021-2022, were compared to incidence rates in season 2019-2020 by calculating incidence rate ratios with 95% CI.

Finally, the relative risk of an RSV case being admitted and an RSV admission leading to an ICT admission was calculated with 95% CI comparing the summer epidemic of season 2021-2022 with the winter season of 2019-2020 because this season was the most comparable regarding testing activity and did not appear significantly shortened by restrictions implemented in March 2020.

For sensitivity analysis, we conducted the same analysis using both the 2017-2018 and 2018-2019 season as reference season.

## RESULTS

The number of persons tested for RSV by season increased from 38 014 in season 2018-2019 to 51 655 in season 2019-2020 decreased in season 2020-2021 to 23 601 and increased markedly to 99 078 in season 2021-2022 (Table 1). The number of RSV cases, RSV-related admissions, and ICT admissions by season were similar for the winter seasons before the COVID-19 pandemic (seasons 2017-2018, 2018-2019, 2019-2020) (Table 1). Because of the absence of RSV in the season 2020-2021, only a few cases were identified, and this season was excluded from further analysis. In the summer epidemic of 2021-2022, the seasonal number of RSV cases, RSV-related admissions, and ICT admissions increased to 11 314 cases, 4103 admissions, and 909 ICT admissions compared with 4,552, 2199, and 452, respectively, in the 2019-2020 season.

**Table 1.** Number of Person-years, Tested Persons, Cases, Admissions, and Patients Receiving ICT by Season, 2017-2018 to 2021-2022

	2017-2018	2018-2019	2019-2020	2020-2021	2021-2022
Person-years	5 715 611	5 739 100	5 741 516	5 885 997	5 800 605
Tested persons	37 239	38 014	51 655	23 601	99 078
RSV cases	4530	4235	4552	<20	11 314
RSV-related admissions	2482	2111	2199	<20	4103
RSV-related ICT admissions	472	452	452	<20	909

Abbreviations: RSV, respiratory syncytial virus; ICT, intensive care treatment.

**Figure 1.**
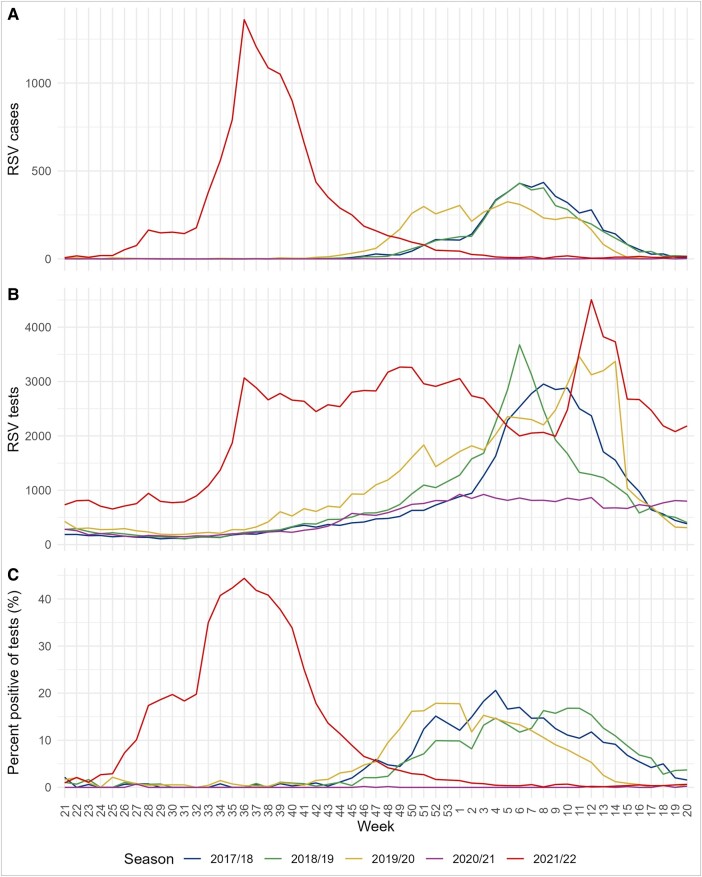
Weekly number of RSV cases (*A*), number of RSV tests (*B*), and percent positive (*C*) by season 2017-2018 to 2021-2022.

Figure 1 shows the weekly number of cases, tests, and the percentage of weekly tests positive for RSV (the percent positive). In the pre-COVID-19 seasons, testing activity for RSV increased as a reaction to the rise in RSV cases, with a peak in weekly tests between 2953 and 3458 tests, and decreased as the number of cases decreased. In the beginning of the summer epidemic, there was a steep increase in the number of weekly tests rising from 785 in week 31 to 3067 in week 36. Unlike previous seasons, the number of tests performed remained high even after the number of RSV cases decreased. The number of weekly tests performed during the weeks with the highest number of RSV cases was comparable across all the seasons, but the percent positive in the summer far exceeded previous winter seasons with 44% in week 36, 2021, compared with 21%, 18%, and 18% for the 3 pre-COVID-19 winter seasons, respectively.

Table 2 shows the incidence rates per 1000 person-years with 95% CI for RSV tests, RSV cases, RSV-related admissions, and ICT admissions stratified by season and age group (see Supplementary Table 2 for the numbers). The test incidence rates per 1000 person-years increased for all age groups during the pre-COVID-19 winter seasons, decreased in the winter season of 2020-2021, and increased in the season of the summer epidemic to a level almost twice as high or more compared with the pre-COVID-19 winter seasons. The test incidence for all seasons was highest in children aged 0 to 2 months, followed by children aged 3 to 5 months and 6 to 23 months. In the summer epidemic test, incidence was also high for children aged 2 to 3 years and people aged 65 years and older with incidence rates of 45.9 (44.7-47.1) per 1000 person-years and 33.4 (33.1-33.7) per 1000 person-years, respectively. Test incidence was lowest in the age groups between 4 and 64 years.

**Table 2.** Incidence Rates With 95% CI of RSV Tests, RSV Cases, RSV-related Admissions, and ICT per 1000 Person-years by Age Group and Season

Season	IR of Tests per 1000 Person-years (95% CI)		IR of Cases per 1000 Person-years (95% CI)		IR of Admissions per 1000 Person-years (95% CI)		IR of ICT per 1000 Person-years (95% CI)	
0-2 mo								
2017-2018	142.5	(136.6-148.7)	53.0	(49.4-56.8)	39.7	(36.6-43.0)	14.1	(12.3-16.1)
2018-2019	155.4	(149.2-161.8)	46.9	(43.5-50.5)	33.9	(31.0-37.0)	13.6	(11.8-15.6)
2019-2020	170.0	(163.5-176.7)	50.9	(47.4-54.6)	35.7	(32.8-38.9)	13.6	(11.8-15.6)
2020-2021	60.3	(56.5-64.3)	…	…	…	…	…	…
2021-2022	382.7	(372.9-392.7)	120.7	(115.2-126.3)	77.8	(73.4-82.3)	30.7	(28.0-33.7)
3-5 mo								
2017-2018	78.8	(74.4-83.4)	36.8	(33.8-40.0)	21.3	(19.0-23.7)	3.0	(2.2-4.0)
2018-2019	85.2	(80.6-89.9)	33.3	(30.4-36.3)	17.6	(15.5-19.9)	3.7	(2.8-4.8)
2019-2020	106.4	(101.2-111.8)	43.5	(40.2-47.0)	20.2	(18.0-22.6)	4.0	(3.1-5.2)
2020-2021	26.6	(24.0-29.3)	…	…	…	…	…	…
2021-2022	191.9	(184.9-199.0)	74.0	(69.7-78.5)	27.2	(24.6-29.9)	5.7	(4.5-7.0)
6-23 mo								
2017-2018	38.3	(37.0-39.6)	14.1	(13.3-14.9)	6.6	(6.1-7.2)	1.1	(.9-1.4)
2018-2019	48.5	(47.1-49.9)	15.6	(14.8-16.4)	7.1	(6.6-7.7)	1.1	(.9-1.3)
2019-2020	54.1	(52.6-55.6)	16.9	(16.1-17.8)	7.2	(6.6-7.7)	1.1	(.9-1.3)
2020-2021	17.2	(16.3-18.0)	…	…	…	…	…	…
2021-2022	132.1	(129.7-134.5)	44.3	(42.9-45.7)	11.5	(10.8-12.3)	1.9	(1.6-2.2)
2-3 y								
2017-2018	11.8	(11.2-12.4)	2.7	(2.4-3.0)	.8	(.7-1.0)	.06	(.02-.12)
2018-2019	15.1	(14.4-15.8)	2.9	(2.6-3.2)	.9	(.7-1.0)	.04	(.01-.10)
2019-2020	16.5	(15.8-17.3)	3.2	(2.9-3.5)	.9	(.8-1.1)	.11	(.06-.19)
2020-2021	4.1	(3.8-4.5)	…	…	…	…	…	…
2021-2022	45.9	(44.7-47.1)	14.7	(14.0-15.4)	3.1	(2.8-3.5)	.51	(.39-.65)
4-5 y								
2017-2018	6.1	(5.6-6.5)	.7	(.5-.8)	.2	(.1-.3)	.03	(.01-.09)
2018-2019	7.2	(6.7-7.7)	.5	(.4-.7)	.1	(.0-.1)	.00	(.00-.03)
2019-2020	9.5	(8.9-10.0)	.8	(.7-1.0)	.2	(.1-.3)	.03	(.01-.08)
2020-2021	2.3	(2.0-2.5)	…	…	…	…	…	…
2021-2022	17.2	(16.5-17.9)	2.8	(2.5-3.1)	.6	(.5-.8)	.13	(.07-.21)
6-14 y								
2017-2018	3.2	(3.1-3.4)	.2	(.1-.2)	.06	(.04-.08)	.00	(.00-.01)
2018-2019	3.1	(3.0-3.3)	.1	(.1-.2)	.02	(.01-.04)	.00	(.00-.01)
2019-2020	4.9	(4.8-5.1)	.1	(.1-.2)	.02	(.01-.04)	.00	(.00-.01)
2020-2021	1.3	(1.2-1.4)	…	…	…	…	…	…
2021-2022	8.5	(8.3-8.8)	.3	(.3-.4)	.07	(.05-.09)	.01	(.00-.02)
15-44 y								
2017-2018	3.8	(3.7-3.9)	.1	(.1-.1)	.04	(.03-.05)	.00	(.00-.01)
2018-2019	4.1	(4.0-4.2)	.1	(.1-.1)	.02	(.01-.03)	.00	(.00-.01)
2019-2020	5.8	(5.7-5.9)	.1	(.1-.1)	.03	(.02-.04)	.00	(.00-.00)
2020-2021	2.5	(2.4-2.5)	…	…	…	…	…	…
2021-2022	10.9	(10.7-11.0)	.3	(.3-.3)	.05	(.05-.07)	.00	(.00-.01)
45-64 y								
2017-2018	5.8	(5.7-5.9)	.2	(.2-.3)	.1	(.1-.1)	.01	(.01-.02)
2018-2019	5.8	(5.7-5.9)	.2	(.2-.2)	.1	(.1-.1)	.01	(.00-.01)
2019-2020	7.9	(7.7-8.0)	.2	(.2-.2)	.1	(.1-.1)	.01	(.00-.01)
2020-2021	3.7	(3.6-3.8)	…	…	…	…	…	…
2021-2022	12.7	(12.5-12.9)	.4	(.3-.4)	.1	(.1-.2)	.01	(.01-.02)
≥65 y								
2017-2018	13.1	(12.9-13.4)	.7	(.6-.7)	.5	(.5-.6)	.06	(.05-.08)
2018-2019	11.3	(11.1-11.5)	.5	(.5-.5)	.3	(.3-.4)	.06	(.05-.07)
2019-2020	16.0	(15.8-16.3)	.4	(.4-.5)	.3	(.3-.4)	.05	(.04-.06)
2020-2021	9.7	(9.5-9.9)	…	…	…	…	…	…
2021-2022	33.4	(33.1-33.7)	.8	(.7-.8)	.6	(.5-.6)	.07	(.06-.09)

Abbreviations: CI, confidence interval; ICT, intensive care treatment; IR, incidence rate; RSV, respiratory syncytial virus.

The RSV case incidence across all seasons was highest for children aged 0 to 2 months followed by children aged 3 to 5 months and children aged 6 to 23 months (Table 2). In the pre-COVID-19 seasons, the case incidence for children aged 0 to 2 months were similar, ranging from 47.0 (43.5-50.5) to 53.0 (49.4-56.8) per 1000 person-years, but in the summer epidemic the incidence rate increased to 120.7 (115.2-126.3). A similar pattern was detected for children aged 3 to 5 months and 6 to 23 months. In the pre-COVID-19 seasons, case incidences for toddlers and preschoolers were low: 2.7 (2.4-3.0) to 3.2 (2.9-3.5) for 2 to 3 year olds and 0.5 (0.4-0.8) to 0.8 (0.7-1.0) for 4 to 5 year olds. In the summer epidemic, case incidence increased to 14.7 (14.0-15.4) and 2.8 (2.5-3.1) for the 2 age groups, respectively. Case incidence was lowest for the age groups between 6 and 64 years across all seasons. Also, for the age group of people aged 65 years and older, the case incidence was also low across all seasons ranging from 0.4 (0.4-0.5) to 0.7 (0.6-0.7) per 1000 person-years in the pre-COVID-19 seasons with an increase to 0.8 (0.8-0.9) in the summer epidemic.

Across all seasons, the incidence rates of RSV-related admissions and ICT admissions were highest for children aged 0 to 2 months, followed by children aged 3 to 5 months and 6 to 23 months (Table 2). The most marked increases in RSV-related admission incidence rates were, as for cases, reported for children aged 2 to 5 years increasing from 0.9 (0.8-1.1) in season 2019-2020 to 3.2 (2.8-3.5) in the summer epidemic for children aged 2 to 3 years, and from 0.2 (0.1-0.3) to 0.7 (0.5-0.8) children aged 4 to 5 years. For older children and adults, the incidence rates increased in the summer epidemic compared with season 2019-2020, but the incidence rates were similar to the incidence rates of season 2017-2018.

When comparing the most recent pre-COVID-19 season (2019-2020) with the season of the summer epidemic, the incidence rate of cases more than doubled in children aged 0 to 2 months with an incidence rate ratio (IRR) of 2.4 (2.2-2.6) (Table 3). The highest IRRs of cases were detected in the age groups of children aged 2 to 3 and 4 to 5 years with IRR 4.6 (4.1-5.2) and 3.3 (2.6-4.2), respectively. For the age groups older than age 5 years, the IRRs varied from 1.8 (1.6-2.0) to 2.6 (2.0-3.4). A similar pattern was seen for IRRs of RSV-related admissions and ICT admissions, where IRRs of RSV-related admissions in the age groups of children aged 2 to 3 years and 4 to 5 years were 3.3 (2.7-4.2) and 3.8 (2.3-6.5), respectively, and IRRs of ICT admissions were 4.5 (2.5-8.7) and 5.0 (1.4-27.0), respectively. Testing activity also increased for all age groups. The increase was highest among children aged 2 to 3 years followed by children aged 6 to 23 months and children aged 0 to 2 months with IRRs of 2.8 (1.7-2.9), 2.4 (2.4-2.5), and 2.3 (2.2-2.4), respectively.

**Table 3.** IRRs With 95% CIs for RSV Cases, RSV-related Admissions, and ICT Admissions, Comparing the Season of the Summer Epidemic 2021-2022 With the Winter Season of 2019-2020

Age Group	IRR of Tests (95% CI)		IRR of RSV Cases (95% CI)		IRR of RSV Admissions (95% CI)		IRR of ICT (95% CI)	
0-2 m	2.25	(2.15-2.36)	2.37	(2.18-2.58)	2.18	(1.96-2.41)	2.26	(1.92-2.68)
3-5 m	1.80	(1.70-1.92)	1.70	(1.54-1.88)	1.35	(1.16-1.57)	1.41	(1.00-1.99)
6-23 m	2.44	(2.36-2.53)	2.61	(2.46-2.77)	1.61	(1.46-1.78)	1.75	(1.36-2.27)
2-3 y	2.78	(2.64-2.92)	4.62	(4.14-5.17)	3.34	(2.71-4.15)	4.48	(2.48-8.67)
4-5 y	1.81	(1.69-1.95)	3.30	(2.63-4.18)	3.77	(2.29-6.51)	5.03	(1.44-26.95)
6-14 y	1.72	(1.64-1.80)	2.61	(2.00-3.44)	2.71	(1.43-5.42)	1.54	(.18-18.39)
15-44 y	1.87	(1.83-1.91)	2.37	(2.04-2.76)	1.84	(1.35-2.54)	1.65	(.32-10.65)
45-64 y	1.62	(1.58-1.65)	1.99	(1.71-2.31)	1.61	(1.28-2.04)	1.68	(.81-3.62)
≥65 y	2.09	(2.05-2.12)	1.83	(1.64-2.05)	1.75	(1.54-2.00)	1.46	(1.03-2.09)

Abbreviations: CI, confidence interval; ICT, intensive care treatment; IRR, incidence rate ratio; RSV, respiratory syncytial virus.

The risk that an RSV case was admitted to hospital in the 2021-2022 season was significantly lower than in the 2019-2020 for the age groups of children aged 0 to 3 years and adults aged 45 to 64 years (Table 4). The incidence risk ratios for children aged 0 to 3 years were between 0.6 (0.6-0.7) in children aged 6 to 23 months and 0.9 (0.9-1.0) in children aged 0 to 2 months; and the incidence risk ratio was 0.8 (0.7-1.0) for people aged 45 to 64 years. For the other age groups, the risk of an RSV case being admitted in the summer epidemic was not significantly different from the winter season of 2019-2020. The risk of an RSV-related admission resulting in an ICT admission was not significantly different in any age groups when comparing the summer epidemic of 2021-2022 with the winter season of 2019-2020 (Table 4).

**Table 4.** Relative Risk With 95% CI of an RSV Case Being Admitted and an Admitted Case Receiving ICT, Comparing the Season of the Summer Epidemic 2021-2022 With the Winter Season of 2019-2020

Age Group	RR of Admissions (95% CI)		RR of ICT (95% CI)	
0-2 m	.92	(.87-.97)	1.04	(.91-1.18)
3-5 m	.79	(.71-.89)	1.05	(.78-1.40)
6-23 m	.62	(.57-.67)	1.09	(.86-1.37)
2-3 y	.72	(.61-.86)	1.34	(.78-2.31)
4-5 y	1.14	(.74-1.77)	1.33	(.43-4.13)
6-14 y	1.04	(.59-1.81)	.57	(.11-3.05)
15-44 y	.78	(.60-1.02)	.90	(.22-3.63)
45-64 y	.81	(.68-.97)	1.04	(.55-1.99)
≥65 y	.96	(.90-1.02)	.83	(.61-1.14)

Abbreviations: CI, confidence interval; ICT, intensive care treatment; RR, relative risk; RSV, respiratory syncytial virus.

Results were similar when comparing seasons 2017-2018 and 2018-2019 as reference seasons (Supplementary Tables 3 and 4).

## DISCUSSION

The timing of the RSV summer epidemic in 2021 was indeed unusual; however, perhaps the most surprising observation was a shift in age groups affected by RSV. The largest increase in case incidences were seen in the age groups of children aged 2 to 3 years and 4 to 5 years with IRRs of 4.6 (4.1-5.2) and 3.3 (2.6-4.2), respectively, when comparing the summer epidemic with the 2019-2020 RSV winter season. For other age groups, the incidence rates were also significantly higher in the summer epidemic than in the pre-COVID-19 winter seasons.

Despite no changes in the official testing policy for RSV during the study period, more people were tested in the surveillance season of the summer epidemic compared with the pre-COVID-19 winter seasons. This was largely driven by increased testing in weeks with no RSV activity and might partly be due to the use of multiplex assays testing for SARS-CoV-2, RSV, and influenza. However, despite an overall increase in testing activity, testing activity during weeks with RSV activity was comparable to pre-COVID-19 winter seasons. Furthermore, the percent positive remained high during the entire summer epidemic, with the highest percent positive of 44%, twice as high as the as the highest recorded percent positive of the pre-COVID-19 winter seasons. For incidence rates of RSV-related admissions, a similar pattern as for cases was detected. It is expected that during the study period, children admitted to a hospital because of severe disease during an RSV epidemic would be tested for RSV; thus, testing activity is thought to affect the number of admissions and intensive care treatment to a lesser extent than number of cases. Taken together, the results indicate that the summer epidemic did exceed the previous winter seasons in magnitude. The shift in age groups was also seen in England, Australia, and the Netherlands [13, 19, 20]. For out-of-season RSV epidemics in other countries, some have reported higher peaks compared with previous winter seasons, whereas others have not [10, 12, 13].

When comparing the summer epidemic with the winter season of 2019-2020, the risk of a case being admitted to a hospital was slightly lower or comparable in all age groups. Also, the risk of an admitted case to be treated with intensive care during admission was comparable across seasons. This suggests that the disease severity spectrum of the 2021 RSV summer epidemic was similar to the usual winter epidemics at population level, as also reported by Nygaard et al [21].

Social restrictions and sanitary measures were implemented in March 2020 to reduce transmission of SARS-CoV-2 [6, 22]. As a consequence, the circulation of the usual respiratory pathogens, including RSV, were greatly reduced [7] causing a longer than usual interval of approximately 60 weeks between the end season of 2019-2020 and the start of the summer epidemic of 2021-2022, which in turn left a large susceptible group of both young and slightly older children. This phenomenon of a prolonged period without exposure has been discussed to cause an immunity debt, potentially leading to higher subsequent epidemics [14, 20, 23, 24]. The effect of restrictions on RSV circulation was observed in several European countries where only Iceland and France experienced a delayed RSV epidemic in the 2020-2021 season [25]. The unusual timing of the summer epidemic was temporally associated with the gradually lifting of restrictions during the spring and early summer in Denmark [26], and similar out-of-season RSV outbreaks were detected in other countries [25, 27].

The increase observed among infants younger than 6 months can, however, not be caused alone by this prolonged period between 2 seasons because these children are immunologically naïve in every season. Infants might be protected by maternal antibodies within the first 6 months of age [28–30]; thus, the increase could therefore be due to reduced levels of maternal antibodies during pregnancy [31]. Another explanation could be that the increased number of RSV cases among older siblings have caused a higher risk of household transmission [32–34].

The incidence rate of RSV-related admissions is by far highest in the group of children aged 0 to 2 months, followed by children aged 3 to 5 months and 6 to 23 months; thus, the consequences associated with an RSV infection is highest among young children. However, the summer epidemic revealed that older children can become severely ill as well, which is important when discussing future vaccine strategies. Prevention of RSV in infancy poses a great opportunity for reducing the overall burden of severe RSV infections and currently vaccines against RSV given to mothers during pregnancy or to infants have been approved or are in trial [35]. However, because the vaccines induce passive and possibly short-lived immunity that potentially interfere with the child's own immune system [30, 36], vaccines might postpone the time for the first infection toward early childhood, thereby increasing the RSV burden in older children as suggested in the modelling study by Zheng et al [37]. On the other hand, an important difference between the pandemic and RSV infant immunization is that available immunization strategies are not expected to decrease transmission in the population and have so far only been proven to protect against lower respiratory tract infections and hospitalizations [38–40].

The high number of tests were also seen in older age groups, but despite an increased number of tests in the adult age groups, the overall incidence rate of cases and RSV-related admissions for adults aged 65 years and older remained low and comparable to the level of the winter season of 2017-2018. The incidence rates of admissions found in this study are comparable to the findings in 2 recent meta-analyses [41, 42] based on pre-COVID-19 winter seasons, though the incidence rates of cases were lower in this study, especially compared with the study by Shi et al. The reason for this might be that adults in Denmark are mainly tested for RSV in relation to an admission. It is unknown why the summer epidemic of 2021 seemed to affect the older adults to a lesser extent than children, but 1 explanation could be that this age group was still careful even after relaxation of restrictions and was therefore protected during this first postpandemic epidemic. This seems likely also based on results from the following season (2022-2023) showing a much larger effect in this age group [43].

This register-based study has some limitations. First, cases were defined as any individual with a positive test for RSV and might therefore include cases and RSV-related admissions where the individual did not present with respiratory symptoms. Using RSV-specific or related diagnosis codes could potentially add valuable clinical information; however, coding practices may change over time or may be used differently in different geographical areas, in different age groups, or at different hospital departments. Second, during the 2021-2022 season, the test pattern for RSV changed markedly, as far more individuals were tested than in previous seasons. It cannot be ignored that part of the increase can be explained by increased testing activity, but during weeks of RSV activity, the testing activity was more comparable across seasons. Third, despite no official change in testing policy, local changes might have been implemented during the study period.

In conclusion, the summer epidemic of 2021 was considerably greater in magnitude than previous winter seasons, among both confirmed cases, admissions and patients that received ICT. Likely because of immunity debt [23] and a larger group of susceptible individuals resulting from COVID-19 restrictions, a shift in age groups affected by RSV was observed, most remarkably among children aged 2 to 5 years, who were 3 to 4 times more likely to be admitted than in previous seasons. Further, there were no indications that the disease severity spectrum of the 2021 RSV summer epidemic was different from the usual winter epidemics.

## Supplementary Material

ofae069_Supplementary_Data

## Supplementary Data

Supplementary materials are available at *Open Forum Infectious Diseases* online. Consisting of data provided by the authors to benefit the reader, the posted materials are not copyedited and are the sole responsibility of the authors, so questions or comments should be addressed to the corresponding author.

## Notes

***Acknowledgments.*** Test results for respiratory syncytial virus (RSV) were obtained from the Danish Microbiology Database (MiBa, http://miba.ssi.dk), which contains all electronic reports from departments of clinical microbiology in Denmark since 2010. We acknowledge the collaboration with the MiBa Board of Representatives.

***Ethics***. This study was performed as a surveillance study under the authority task of Statens Serum Institut with administrative register data. According to Danish law, ethical approval is not needed for this type of research.

***Consent to participate***. This study does not include factors necessitating patient consent.

***Previous presentations of the work***. Some of the results presented in this this manuscript were presented at an oral session at the 33rd European Congress of Clinical Microbiology and Infectious Diseases—ECCMID 2023. Abstract number 01118.

***Financial support.*** Neither Statens Serum Institut nor any authors received external funding for this study.
