# Supplementary material for: Resurgence of Respiratory Syncytial Virus in the Summer of 2021 in Denmark—a Large out-of-season Epidemic Affecting Older Children
Source: Open Forum Infect Dis. 2024 Feb 5;11(3):ofae069. doi: 10.1093/ofid/ofae069 (PMC10941316; doi:10.1093/ofid/ofae069)
Supplement: ofae069_Supplementary_Data [file ofae069_supplementary_data.docx]

# Supplementary

## Person time calculation

Person-time was calculated for each individual in each age group for each season. Cases contribute with person-time from either start of season, when the person enters the specific age group or at immigration. Person-time ends either at the end of season if the person becomes a case in that season, if the person exits the specific age group, if the person dies or at emigration, see supplementary figure 1 for an illustration.

Supplementary Figure 1. Illustration of person-time calculation


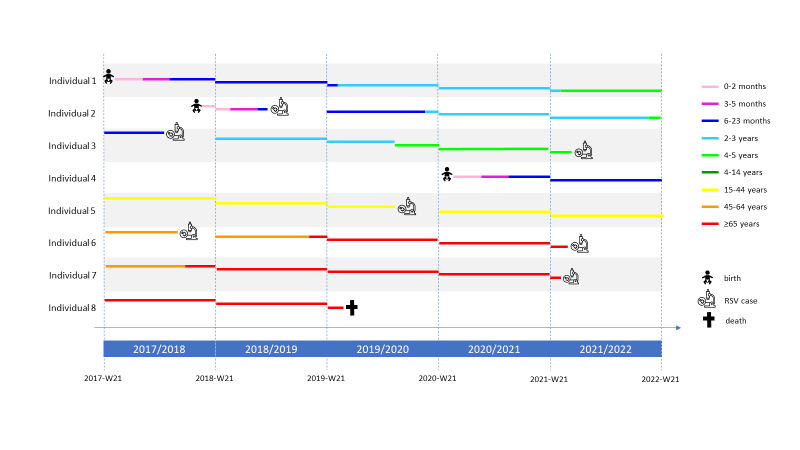


## Tables

Table S1. Table of procedure codes used to define intensive care treatment

| Procedure code | Description |
| --- | --- |
| NABB | Intensive care therapy |
| NABE | Intensive care observation |
| BGDA0 | Mechanical ventilation |
| BGDA1 | Non-invasive ventilation (NIV) |
| BGFC32 | Continuous positive airway pressure therapy (CPAP) |
| BGFC33 | Intermittent CPAP |

Table S2. Number of RSV cases, RSV-related admissions, and ICT admissions by season and age group

| **Age group** | | **2017/2018** | **2018/2019** | **2019/2020** | **2020/2021** | **2021/2022** |
| --- | --- | --- | --- | --- | --- | --- |
| **Tested persons** | | | | | | |
|  | *0-2 months* | 1,998 | 2,112 | 2,360 | 844 | 5,263 |
|  | *3-5 months* | 1,109 | 1,181 | 1,476 | 357 | 2,649 |
|  | *6-23 months* | 3,147 | 3,912 | 4,360 | 1,385 | 10,343 |
|  | *2-3 years* | 1,176 | 1,581 | 1,815 | 417 | 5,036 |
|  | *4-5 years* | 624 | 717 | 993 | 237 | 1,912 |
|  | *6-14 years* | 1,745 | 1,665 | 2,666 | 633 | 4,386 |
|  | *15-44 years* | 7,493 | 8,069 | 11,524 | 4,834 | 21,006 |
|  | *45-64 years* | 7,384 | 7,485 | 10,229 | 4,760 | 15,767 |
|  | *65+ years* | 12,450 | 11,154 | 16,032 | 9,943 | 32,143 |
| **Cases** | | | | | | |
|  | *0-2 months* | 805 | 704 | 768 | - | 1,833 |
|  | *3-5 months* | 553 | 501 | 649 | - | 1,111 |
|  | *6-23 months* | 1,302 | 1,439 | 1,548 | - | 3,955 |
|  | *2-3 years* | 311 | 347 | 392 | - | 1,790 |
|  | *4-5 years* | 78 | 61 | 98 | - | 343 |
|  | *6-14 years* | 93 | 78 | 78 | - | 199 |
|  | *15-44 years* | 274 | 224 | 250 | - | 597 |
|  | *45-64 years* | 365 | 311 | 270 | - | 541 |
|  | *65+ years* | 749 | 570 | 499 | - | 945 |
| **Admissions** | | | | | | |
|  | *0-2 months* | 604 | 509 | 539 | - | 1,181 |
|  | *3-5 months* | 320 | 265 | 301 | - | 408 |
|  | *6-23 months* | 611 | 661 | 656 | - | 1,031 |
|  | *2-3 years* | 98 | 103 | 116 | - | 383 |
|  | *4-5 years* | <20 | <20 | 20 | - | 80 |
|  | *6-14 years* | 33 | 14 | 14 | - | 37 |
|  | *15-44 years* | 82 | 41 | 64 | - | 119 |
|  | *45-64 years* | 146 | 118 | 120 | - | 195 |
|  | *65+ years* | 569 | 391 | 369 | - | 669 |

Table S3. Incidence rate ratios with 95% confidence intervals for RSV cases, RSV related admissions and ICT admissions, comparing the season of the summer epidemic 2021/22 with different pre-COVID-19 winter seasons

| Reference season | | IRR of tests  (95% CI) | | IRR of cases  (95% CI) | | IRR of admissions (95% CI) | | IRR of ICT  (95% CI) | |
| --- | --- | --- | --- | --- | --- | --- | --- | --- | --- |
| 0-2 months | | | | | | | | | |
|  | *2017/18* | 2.69 | (2.56; 2.82) | 2.28 | (2.10; 2.48) | 1.96 | (1.77; 2.16) | 2.18 | (1.85; 2.58) |
|  | *2018/19* | 2.46 | (2.35; 2.58) | 2.57 | (2.36; 2.81) | 2.29 | (2.06; 2.55) | 2.26 | (1.91; 2.68) |
|  | *2019/20* | 2.25 | (2.15; 2.36) | 2.37 | (2.18; 2.58) | 2.18 | (1.96; 2.41) | 2.26 | (1.92; 2.68) |
| 3-5 months | | | | | | | | | |
|  | *2017/18* | 2.43 | (2.27; 2.61) | 2.01 | (1.82; 2.23) | 1.28 | (1.10; 1.48) | 1.89 | (1.30; 2.78) |
|  | *2018/19* | 2.25 | (2.11; 2.41) | 2.22 | (2.00; 2.48) | 1.54 | (1.32; 1.81) | 1.55 | (1.09; 2.22) |
|  | *2019/20* | 1.80 | (1.70; 1.92) | 1.70 | (1.54; 1.88) | 1.35 | (1.16; 1.57) | 1.41 | (1.00; 1.99) |
| 6-23 months | | | | | | | | | |
|  | *2017/18* | 3.45 | (3.32; 3.58) | 3.14 | (2.95; 3.35) | 1.74 | (1.58; 1.93) | 1.65 | (1.28; 2.13) |
|  | *2018/19* | 2.72 | (2.63; 2.82) | 2.84 | (2.68; 3.02) | 1.61 | (1.46; 1.78) | 1.70 | (1.32; 2.20) |
|  | *2019/20* | 2.44 | (2.36; 2.53) | 2.61 | (2.46; 2.77) | 1.61 | (1.46; 1.78) | 1.75 | (1.36; 2.27) |
| 2-3 years | | | | | | | | | |
|  | *2017/18* | 3.89 | (3.67; 4.13) | 5.53 | (4.90; 6.25) | 3.75 | (3.00; 4.73) | 8.51 | (3.89; 22.03) |
|  | *2018/19* | 3.04 | (2.88; 3.21) | 5.12 | (4.56; 5.76) | 3.69 | (2.96; 4.64) | 12.31 | (5.00; 39.25) |
|  | *2019/20* | 2.78 | (2.64; 2.92) | 4.62 | (4.14; 5.17) | 3.34 | (2.71; 4.15) | 4.48 | (2.48; 8.67) |
| 4-5 years | | | | | | | | | |
|  | *2017/18* | 2.83 | (2.60; 3.08) | 4.20 | (3.28; 5.45) | 4.02 | (2.42; 7.03) | 3.82 | (1.23; 15.71) |
|  | *2018/19* | 2.40 | (2.21; 2.60) | 5.29 | (4.02; 7.07) | 8.37 | (4.19; 18.96) | - | - |
|  | *2019/20* | 1.81 | (1.69; 1.95) | 3.30 | (2.63; 4.18) | 3.77 | (2.29; 6.51) | 5.03 | (1.44; 26.95) |
| 6-14 years | | | | | | | | | |
|  | *2017/18* | 2.63 | (2.49; 2.77) | 2.25 | (1.75; 2.9) | 1.18 | (0.72; 1.94) | 1.57 | (0.18; 18.85) |
|  | *2018/19* | 2.73 | (2.58; 2.88) | 2.65 | (2.03; 3.49) | 2.75 | (1.45; 5.50) | 1.56 | (0.18; 18.68) |
|  | *2019/20* | 1.72 | (1.64; 1.80) | 2.61 | (2.00; 3.44) | 2.71 | (1.43; 5.42) | 1.54 | (0.18; 18.39) |
| 15-44 years | | | | | | | | | |
|  | *2017/18* | 2.88 | (2.81; 2.95) | 2.17 | (1.87; 2.51) | 1.44 | (1.08; 1.94) | 0.99 | (0.23; 4.32) |
|  | *2018/19* | 2.65 | (2.59; 2.71) | 2.65 | (2.27; 3.11) | 2.89 | (2.01; 4.23) | 1.00 | (0.23; 4.32) |
|  | *2019/20* | 1.87 | (1.83; 1.91) | 2.37 | (2.04; 2.76) | 1.84 | (1.35; 2.54) | 1.65 | (0.32; 10.65) |
| 45-64 years | | | | | | | | | |
|  | *2017/18* | 2.18 | (2.13; 2.24) | 1.46 | (1.28; 1.67) | 1.32 | (1.06; 1.64) | 1.14 | (0.59; 2.23) |
|  | *2018/19* | 2.19 | (2.14; 2.25) | 1.72 | (1.50; 1.99) | 1.64 | (1.29; 2.07) | 1.68 | (0.81; 3.62) |
|  | *2019/20* | 1.62 | (1.58; 1.65) | 1.99 | (1.71; 2.31) | 1.61 | (1.28; 2.04) | 1.68 | (0.81; 3.62) |
| 65+ years | | | | | | | | | |
|  | *2017/18* | 2.54 | (2.49; 2.59) | 1.19 | (1.08; 1.31) | 1.1 | (0.99; 1.24) | 1.08 | (0.78; 1.51) |
|  | *2018/19* | 2.97 | (2.91; 3.03) | 1.58 | (1.43; 1.76) | 1.63 | (1.44; 1.86) | 1.18 | (0.85; 1.66) |
|  | *2019/20* | 2.09 | (2.05; 2.12) | 1.83 | (1.64; 2.05) | 1.75 | (1.54; 2.00) | 1.46 | (1.03; 2.09) |

Abbreviations: RSV, respiratory syncytial virus; ICT, intensive care treatment; IRR, incidence rate ratio; CI, confidence interval.

Table S4. Relative risk with 95% confidence interval of an RSV case being admitted and an admitted case receiving intensive care treatment, comparing the season of the summer epidemic 2021/22 with different pre-COVID-19 winter seasons

| Reference season | | RR of admissions (95% CI) | | RR of ICT (95% CI) | |
| --- | --- | --- | --- | --- | --- |
| 0-2 months | | | | | |
|  | *2017/18* | 0.86 | (0.81; 0.90) | 0.86 | (0.81; 0.90) |
|  | *2018/19* | 0.89 | (0.84; 0.94) | 0.89 | (0.84; 0.94) |
|  | *2019/20* | 0.92 | (0.87; 0.97) | 0.92 | (0.87; 0.97) |
| 3-5 months | |  |  |  |  |
|  | *2017/18* | 0.63 | (0.57; 0.70) | 0.63 | (0.57; 0.70) |
|  | *2018/19* | 0.69 | (0.62; 0.78) | 0.69 | (0.62; 0.78) |
|  | *2019/20* | 0.79 | (0.71; 0.89) | 0.79 | (0.71; 0.89) |
| 6-23 months | | | | | |
|  | *2017/18* | 0.56 | (0.51; 0.60) | 0.56 | (0.51; 0.60) |
|  | *2018/19* | 0.57 | (0.53; 0.61) | 0.57 | (0.53; 0.61) |
|  | *2019/20* | 0.62 | (0.57; 0.67) | 0.62 | (0.57; 0.67) |
| 2-3 years | | | | | |
|  | *2017/18* | 0.68 | (0.56; 0.82) | 0.68 | (0.56; 0.82) |
|  | *2018/19* | 0.72 | (0.60; 0.87) | 0.72 | (0.60; 0.87) |
|  | *2019/20* | 0.72 | (0.61; 0.86) | 0.72 | (0.61; 0.86) |
| 4-5 years | | | | | |
|  | *2017/18* | 0.96 | (0.62; 1.48) | 0.96 | (0.62; 1.48) |
|  | *2018/19* | 1.58 | (0.84; 2.98) | 1.58 | (0.84; 2.98) |
|  | *2019/20* | 1.14 | (0.74; 1.77) | 1.14 | (0.74; 1.77) |
| 6-14 years | | | | | |
|  | *2017/18* | 0.52 | (0.35; 0.78) | 0.52 | (0.35; 0.78) |
|  | *2018/19* | 1.04 | (0.59; 1.81) | 1.04 | (0.59; 1.81) |
|  | *2019/20* | 1.04 | (0.59; 1.81) | 1.04 | (0.59; 1.81) |
| 15-44 years | | | | | |
|  | *2017/18* | 0.67 | (0.52; 0.85) | 0.67 | (0.52; 0.85) |
|  | *2018/19* | 1.09 | (0.79; 1.50) | 1.09 | (0.79; 1.50) |
|  | *2019/20* | 0.78 | (0.60; 1.02) | 0.78 | (0.60; 1.02) |
| 45-64 years | | | | | |
|  | *2017/18* | 0.90 | (0.76; 1.07) | 0.90 | (0.76; 1.07) |
|  | *2018/19* | 0.95 | (0.79; 1.14) | 0.95 | (0.79; 1.14) |
|  | *2019/20* | 0.81 | (0.68; 0.97) | 0.81 | (0.68; 0.97) |
| 65+ years | | | | | |
|  | *2017/18* | 0.93 | (0.88; 0.99) | 0.93 | (0.88; 0.99) |
|  | *2018/19* | 1.03 | (0.96; 1.11) | 1.03 | (0.96; 1.11) |
|  | *2019/20* | 0.96 | (0.90; 1.02) | 0.96 | (0.90; 1.02) |

Abbreviations: RSV, respiratory syncytial virus; ICT, intensive care treatment; RR, relative risk; CI, confidence interval.
